# Capturing Workplace Gossip as Dynamic Conversational Events: First Insights From Care Team Meetings

**DOI:** 10.3389/fpsyg.2021.725720

**Published:** 2021-10-12

**Authors:** Vanessa Begemann, Svea Lübstorf, Annika Luisa Meinecke, Frank Steinicke, Nale Lehmann-Willenbrock

**Affiliations:** ^1^Department of Industrial/Organizational Psychology, Institute of Psychology, University of Hamburg, Hamburg, Germany; ^2^Department of Informatics, University of Hamburg, Hamburg, Germany

**Keywords:** workplace gossip, meetings, interaction dynamics, behavioral annotation, elderly care teams

## Abstract

Even though gossip is a ubiquitous organizational behavior that fulfils important social functions (e.g., social bonding or emotion venting), little is known about how workplace gossip and its functions unfold *in situ*. To explore the dynamic nature and social embeddedness of workplace gossip, we develop a behavioral annotation system that captures the manifold characteristics of verbal gossip behavior, including its valence and underlying functions. We apply this system to eight elderly care team meetings audio- and videotaped in the field, yielding a sample of *N* = 4,804 annotated behaviors. On this empirical basis, we provide first insights into the different facets and functions of workplace gossip in real-life team interactions. By means of lag sequential analysis, we quantify gossip patterns that point to the temporal and structural embeddedness of different types of workplace gossip expressions. Though exploratory, these findings help establish workplace gossip as a dynamic conversational event. We discuss future interdisciplinary research collaborations that behavioral observation approaches offer.

## Introduction

*A co-worker leaves in the middle of the meeting. The following conversation unfolds: “He arrived half an hour late, but leaves on time.” - “But he didn’t know.” - “Instead of half past six, he arrived at seven.” - “Yeah, true*…*“ - “You don’t just walk out of a meeting.”*

We are probably all familiar with these types of conversations at work. Frequently, we can catch ourselves overhearing or engaging in evaluative comments about someone who is not present – in short, we gossip (Foster, 2004). Aside from the infamous watercooler, workplace gossip can happen almost anywhere: in the hallway, kitchen, elevator, and even in more formal settings such as meetings (Hallett et al., 2009). Studies suggest that gossip accounts for nearly 14% of our conversations (Robbins and Karan, 2020), and more than 90% of the workforce engages in gossip (Grosser et al., 2012).

Although gossip is a ubiquitous communication behavior, it usually has a negative reputation with many organizational studies focusing on its negative consequences (e.g., Kuo et al., 2015; Tan et al., 2020). As a result, organizational scholars and management consultants regularly recommend reducing or preventing gossip at work (e.g., Riegel, 2018; Wu et al., 2018; Heathfield, 2019). Despite its negative reputation, however, gossip serves important social functions such as group protection, emotion venting, or entertainment (Baumeister et al., 2004; Foster, 2004; Grosser et al., 2012). Moreover, gossip does not necessarily have to be negative in nature. Recent conceptualizations state that gossip can comprise both negative and positive talk about absent third parties (Brady et al., 2017; Lee and Barnes, 2021). This is described as *valence*, the positivity or negativity of the information that is shared.

Scholars agree that gossip is strongly influenced by the context (Mills, 2010) and has a variety of facets (Lee and Barnes, 2021). Like most phenomena in workplace interactions, workplace gossip is embedded in a dynamic social context and emerges over time (cf. Lehmann-Willenbrock and Allen, 2018). However, studies that observe actual gossip behavior *in situ* are scarce. Instead, most extant work is based on self-reported survey data (e.g., Kuo et al., 2015; Brady et al., 2017; Kong, 2018). Although previous self-report studies have offered important insights into the relationship of gossip with other variables (e.g., work performance; Tian et al., 2019), they come with several challenges. First, self-report surveys of gossip provide static snapshots rather than insights into the conversational context in which gossip occurs. Second, they are not able to capture the delicate nuances of workplace gossip, such as sudden changes in valence within a gossip conversation. Similarly, throughout a conversation, workplace gossip may serve multiple social functions that may change depending on the content. Third, self-reports have the risk of being biased, in terms of social desirability and memory effects when relying on reports about behavioral intentions or past behavior (e.g., Baumeister et al., 2007). Taken together, there is a considerable research gap regarding gossip behavior as dynamic and conversational events embedded in communicative context. Thus, it is crucial to also consider its temporal interaction dynamics by means of behavioral observation (Lehmann-Willenbrock and Allen, 2018).

Health care offers a suitable context to study workplace gossip *in situ* (Kim et al., 2021). In service-oriented jobs such as health care, gossip is often an inherent part of the job as nurses need to exchange information about their patients to fulfill their work tasks (Nübling et al., 2010). Regular team meetings, as a core interactional context in organizations, offer a platform to exchange such information (Lehmann-Willenbrock et al., 2018). As a research setting in general, team meetings can be used as a gateway to study team interaction processes and complex communication dynamics (Meinecke and Lehmann-Willenbrock, 2015). Thus, to empirically investigate our research questions, in the current study we turn to regular face-to-face team meetings from the health care sector.

In sum, the current study builds initial knowledge about workplace gossip as dynamic conversational events. In particular, we explore how the valence and functions of workplace gossip unfold during conversations by investigating a sample of audio- and video-recorded meetings of elderly care teams. We analyze their verbal behavior by annotating every single gossip event according to its valence and functions. As such, we offer the following contributions. First, we map and study workplace gossip as a dynamic conversational event, with fluctuations in valence within the flow of communication. By capturing and annotating workplace gossip behavior unfolding during regular workplace meetings in the healthcare context, our study reveals the nuances of different types of behavioral gossip expressions. Our exploratory insights suggest that workplace gossip serves several important social functions, depending on the valence of the gossip. Second, our study provides insights into the temporal embeddedness of different gossip types regarding their valence, based on a lag sequential analysis that uncovers behavioral patterns surrounding different types of gossip behavior. Finally, we discuss ideas for future interdisciplinary collaborations between social scientists and computer scientists.

## Theoretical Background

### The Many Facets and Functions of Workplace Gossip

Gossip is a fundamentally social communicative behavior that includes at least three people, i.e., the sender, the receiver, and the absent target (Dunbar, 2004). Since gossip is central to the human behavioral repertoire and most adults spend a significant amount of their time at work, gossip also occurs in the workplace (Grosser et al., 2012). In defining workplace gossip for the current study, we follow Brady et al. (2017) who conceptualize workplace gossip as “informal and evaluative (i.e., positive or negative) talk from one member of an organization to one or more members of the same organization about another member of the organization who is not present to hear what is said” (Brady et al., 2017, p. 3). Two aspects are central to this definition—gossip is informal and evaluative in nature.

As a first defining feature, the informal nature of gossip denotes that gossip usually happens outside of organizational control. That is, gossip is informal communication and is often said to be part of the organizational grapevine (Beersma and van Kleef, 2011; Grosser et al., 2012). The grapevine refers to the unofficial, informal communication network in organizations through which information can be shared quickly between members of the organization. This informal nature of gossip also shapes the common image of the so-called watercooler talk that many have in mind when they think of workplace gossip. In other words, gossip tends to be associated with confidential conversations among trusted co-workers. Yet, ethnographic studies have shown that this is not necessarily always the case. Although gossip can be categorized as informal talk, gossip can also occur in more formal settings such as regular team meetings. Applying linguistic ethnography, Hallett et al. (2009) observed that workplace gossip is a common and reoccurring communication event during regular school staff meetings. The same applies to meetings in university contexts (Carrim, 2016).

These prior findings indicate that gossip constitutes a complex and delicate form of verbal exchange. Whether and how someone gossips largely depends on the context people find themselves in. Gossip usually is not simply blurted out but is rather embedded in the flow of communication between two or more conversational partners. For example, subtle cues may invite a person to make an evaluative statement about someone not present. Depending on the reaction of the gossip recipient(s), the gossip may then further spread in the group so that everyone contributes something evaluative to the conversation.

The second key defining feature of workplace gossip concerns its evaluative nature. Gossip is typically considered to be either positive or negative in valence. An example of positive gossip is praising a coworker’s performance at work, whereas an example of negative workplace gossip would be questioning someone’s morale (Brady et al., 2017; Lee and Barnes, 2021). Previous research has found contrasting effects for positive and negative gossip. For example, in a study of employee-supervisor dyads across different industries, Kuo et al. (2018) found that whereas positive supervisor workplace gossip significantly improved the relationship between the supervisor and employee, negative supervisor workplace gossip had no effect. Other studies, however, have found no difference between positive and negative gossip and important outcome variables (e.g., performance ratings; Grosser et al., 2010). These mixed findings suggest that the effects of gossip are significantly more complex than initially assumed (Lee and Barnes, 2021). In this regard, the assumed dichotomy between positive and negative gossip has received criticism. Some scholars argue that gossip need not be evaluative at all, as long as it centers on talk about an absent target (Dores Cruz et al., 2021). Support for this view was provided by a recent naturalistic observation study. By applying electronically activated recording devices, Robbins and Karan (2020) found that most everyday gossip is neutral in nature.

Another important stream in the literature on (workplace) gossip is dedicated to the manifold social functions that are associated with gossip. Much of this research is motivated by the fact that despite its negative reputation and possible negative consequences, people tend to gossip on a regular basis (Dunbar, 2004; Grosser et al., 2012). Typical functions associated with gossip are the following. First, people are motivated to gossip because it allows them to share and validate information with one another (Foster, 2004; Grosser et al., 2012). For example, gossip can be used to gather information about a person with whom one has not (yet) had much contact. This can help individuals form an opinion about that person or compare their own opinion with that of trusted coworkers. Second, workplace gossip can help to establish and maintain important group norms and values (Baumeister et al., 2004; Dunbar, 2004). For example, new employees can socialize through gossip and become familiar with group norms and organizational culture (Laing, 1993; Chase and Stuart, 1995). Third, gossip can be used as group protection to warn other in-group members about free-riders or other unpleasant encounters (Beersma and van Kleef, 2012). This way, in-group members can, for example, avoid working with certain individuals in the future which can prevent them from being taken advantage of. Fourth, workplace gossip can also function as entertainment (Foster, 2004; Grosser et al., 2012). Especially in monotonous work environments, gossip can serve as a form of pleasant distraction. Fifth, gossip can be used as an outlet for emotions (Dores Cruz et al., 2019). As such, gossip can serve as a short-term emotion-focused coping style to deal with stress and negative emotions (Waddington, 2005). Finally, workplace gossip can be used to negatively influence the recipient’s opinion of the target (Beersma and van Kleef, 2012; Dores Cruz et al., 2019), for example, to damage the target’s reputation or to increase their own status. These six functions are not mutually exclusive but can occur simultaneously in any gossip exchange (Grosser et al., 2012; Beersma et al., 2019).

### Observing Workplace Gossip *in situ*

An important research gap concerns the question how gossip senders and recipients shift between more positive and negative instances of workplace gossip. Previous research largely looked at gossip in general and as isolated events, assuming that the exchange focused on either positive or negative content (e.g., Kuo et al., 2018; Ben-Hador, 2019). In reality, however, gossip is embedded in the flow of communication and individuals are usually driven by a certain ambivalence. Hence, we assume that conversational actors will switch between positive and negative gossip within a sequence of gossip statements.

Furthermore, our current knowledge of gossip in the workplace is still very limited regarding the link between valence and functions. Past research has mostly relied on self-reports to capture the functions underlying gossip (e.g., Dores Cruz et al., 2019). Such self-reports are susceptible to various biases and may lead to socially desirable responses (Turner et al., 2003). For example, it is unclear whether only negative gossip is associated with group-serving functions such as the establishment of group norms and values, or whether only positive gossip serves as entertainment and is associated with shared laughter.

To address these knowledge gaps, we build on previous theoretical work (Mills, 2010) and argue that workplace gossip should be seen and ultimately studied as a highly context-dependent and multi-faceted phenomenon. In order to better understand the nuances of gossip, the question now arises as to the appropriate context in which to examine gossip *in situ*. As a research setting for studying workplace gossip, we focus on team meetings (cf. Hallett et al., 2009; Carrim, 2016). Observing actual gossip behavior in real life meetings provides an opportunity to gain a much richer understanding of the phenomenon in the field (Kolbe and Boos, 2019). Specifically, we investigate gossip during elderly care team meetings.

### Workplace Gossip in Health Care Settings

Nurses’ daily job duties rely on interdependent collaboration, and therefore teamwork is very common in the healthcare sector (Dinh et al., 2020). A substantial part of a nurse’s job is interacting and communicating with others (Kerr, 2002), which includes sharing information about others who may be absent (e.g., patients, physicians, or other nurses). In fact, sharing critical information about others is even formally organized through regular hand-over meetings between shifts (Tobiano et al., 2020). Following the reasoning of Babalola et al. (2019), these circumstances (i.e., high interaction intensity) should favor the occurrence of gossip. Indeed, previous research found that gossip is associated with an interest in people-oriented professions such as nursing (Nevo et al., 1993).

Additionally, high levels of stress are common in health care (Zhang et al., 2014). Time pressure and a lack of available resources are just two of many factors that contribute to high stress levels among nurses (Testad et al., 2010). Positions for professionals remain vacant for around 175 days (Bonin, 2020), leaving most care institutions understaffed and increasing the workload for those who remain. Consequently, to deal with such a stressful work environment, nurses were found to use workplace gossip to share negative emotions and cope with stress (Waddington and Fletcher, 2005; Thomas and Rozell, 2007; Altuntaş et al., 2014; Jiang et al., 2019). In sum, we believe that these characteristics of the health care context give rise to a variety of workplace gossip behavior, making health care settings a suitable context to study gossip at work.

Taken together, the current study turns to regular team meetings in health care to explore workplace gossip as a complex conversational construct that is embedded in the flow of communication. In particular, we investigate the dynamic nature of the valence and functions of workplace gossip within conversations. The following research questions guide our efforts:

*(1) How does the valence of workplace gossip unfold in the flow of team communication?*
*(2) How do the social functions and valence of gossip statements interact in team meetings?*

## Method

The current study was part of a larger research project that was approved by the local ethics committee at the University of Hamburg (title: Team dynamics in stationary care teams). Participation was voluntary, subject to informed consent, and all participants retained the right to opt out of data gathering at any point.

### Participants and Procedure

We recruited eight nursing teams working in four different elderly care homes in Germany. All elderly care homes belonged to the same organization. Team sizes ranged from six to ten members, which is in line with team sizes reported in previous studies of teams in stationary care (Moser et al., 2019). A total of *N* = 62 nurses were observed. The majority of our participants (90.16%) were female, reflecting the gender distribution in the health care sector in general (e.g., Neff et al., 2011).

Prior to recording the team meetings, the teams were informed of the overall study purpose and procedure and provided their written informed consent. Each team leader was contacted via telephone to obtain further information about the observation procedure and, in case of consent, to schedule a date for the observation. As an incentive for participation, we offered detailed feedback on the teams’ behavior during the meeting.

**FIGURE 1:**
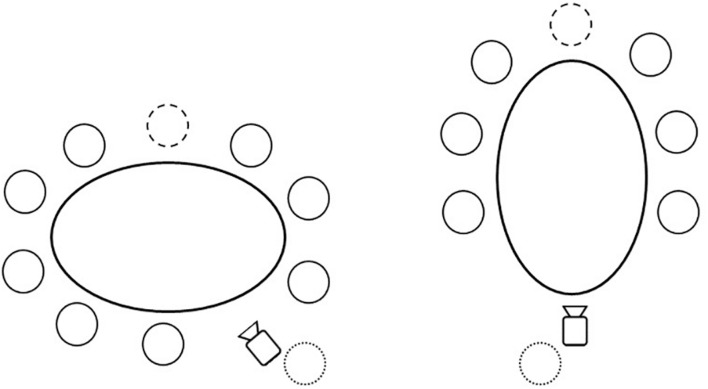
Illustration of two usual set-ups of the meeting recordings including the camera location. Small circles illustrate persons; the circle with the dotted line behind the camera illustrates the experimenter; the circle with the dashed line at the top of the table illustrates team leader.

Participants were instructed to conduct their meeting as usual and to ignore the camera. The camera with built-in microphone was located at one end of the meeting table and was used to audio- and videotape every meeting member (see Figure 1 for the schematic setup). We acknowledge that the decision to use a single camera/microphone to record a group comes with impaired signal quality (e.g., when more than one person speaks at the same time). We chose to observe the gossip behavior in a more naturalistic setting. Thus, we wanted to keep the influence by a salient study setup (e.g., a camera and separate microphone for each meeting member) on the team members’ usual meeting behavior to a minimum. As soon as the meeting ended, the recording was stopped. To address potential social desirability bias, we asked the participants afterward whether the recorded meeting differed from their usual team meetings, which was not the case for any of the observed meetings. We audio- and video-recorded one meeting of each team, resulting in eight recorded team meetings.

### Setting: Monthly Team Meetings

On a monthly basis, the team leader of each observed team schedules and prepares a meeting during which all team members are present, unless they have to care for the patients. The participating teams were hierarchically organized, with the head nurse leading the meeting. All observed team meetings included one or two skilled nurses, two to three care assistants, and one service worker. The meeting usually starts with the team leader briefly presenting the agenda and asking about the current mood of the team members. The monthly team meetings provide a platform to share important information about current work issues, ranging from information about patients and colleagues to general information about work procedures and regularities. The team leader also uses this meeting to give work-related feedback to the staff, provide work instructions, and plan future work schedules with the team. The team members use the monthly meetings to raise concerns about current work issues, ask questions about how to handle difficult situations, or voice complaints. As many of the team members do not see each other on a regular basis due to shift work, the meetings also provide an opportunity to talk with most of the team at the same time and to socialize. These general characteristics apply to all eight meetings. Nonetheless, the meetings somewhat differed concerning the composition of the meeting attendees. For example, in three meetings, the nursing manager was present as well. Furthermore, as teams discussed current work issues and other agenda items relevant for their respective team, the actual content of the meetings was unique for each meeting (e.g., one meeting was recorded in December and included long discussions about the upcoming Christmas party).

### Characteristics of Initial Speech Material

Although we were only interested in the verbal behavior, we still used video-recordings, as the segmentation of the speakers and anticipation of speaker turns can be challenging otherwise (particularly in larger teams and in field settings where the audio signal quality may not be ideal). The video data including audio for each meeting was stored as an mp4-file.

We recorded a total of 323 minutes of meeting behavior. Meeting length varied considerably, ranging from 17 to 73 minutes (*M* = 43.63, *SD* = 21.36). The conversational shares (i.e., the percentage for each team member of their verbal participation in the meeting, based on duration) ranged from 0.18 to 74.24% (*M* = 11.43, *SD* = 18.47). This includes conversational shares of everyone, including the team leader and the special case of every team member speaking at the same time. When looking at the conversational shares of the team leaders only, the percentages range from 46.86 to 74.24% (*M* = 59.63, *SD* = 11.04). The percentages of times in which multiple team members talked at the same time ranged from 1.11 to 5.24% (*M* = 2.57, *SD* = 1.47). Speaker turns per meeting (i.e., how often the role of the speaker switches from one team member to another) ranged from 127 to 687 (*M* = 411.96, *SD* = 175.78).

### Gossip Coding Scheme

To annotate gossip behavior in the meetings, we developed a coding scheme using an iterative process in which we refined the coding manual and annotation approach several times in response to feedback from the raters (e.g., providing more precise examples). We describe the behavioral categories and codes used for the annotation process in detail below. An overview including sample statements is presented in Table 1.

**TABLE 1:** Annotation system for verbal gossip events.

Dimension	Coding category			
Valence	**Positive** Content of statement is positive *“But she can trust herself to do that, because professionally she’s not bad.”*	**Negative** Content of statement is negative *“But the problem here is that he makes appointments with people and just doesn’t show up at all.”*	**Ambiguous** Content of statement is ambiguous (i.e., can be both positive and negative) *“And then, of course, it happened to him, right.”*	**Neutral** Content of statement is neutral *“She has to request a new prescription from the doctor.”*

Function	**Information** Sharing and validating information *“Sometimes I feel like she doesn’t understand - language-wise.”*	**Group norms and values** Establishing and maintaining group norms and values *“She arrived half an hour late and is now leaving.”*	**Group protection** Protecting group from free-riders and other unpleasant encounters “*Sometimes she says ‘Yes, yes, I know, I understand’, but then does nothing.”*	
	**Entertainment** Pleasant and fun exchange “*[…] they stole her pants.*”	**Emotion venting** Coping with negative emotions *“She criticizes everything!”*	**Negative influence** Influencing others’ opinion about the target in a negative way *“[*…*] he wasn’t there. Where, for example, I was there in person and could say, no, there was no on there.”*	

*Behavioral codes are written in bold, examples are in italics.*

#### Valence

Valence was assessed with a set of four mutually exclusive categories. That is, each gossip event was assigned exactly one out of four valence categories. Based on traditional definitions of workplace gossip, we differentiated between positive and negative gossip. As the line between positive and negative gossip can be blurred at times, we also included a third category for ambiguous gossip. Ambiguous gossip was annotated when a statement was sarcastic or when the valence of the literal statement and the tone of voice did not match. Finally, and in line with findings by Robbins and Karan (2020), we further decided to include neutral gossip as a fourth category.

#### Functions

Gossip can serve several functions at the same time (Grosser et al., 2012; Beersma et al., 2019). Thus, our categories for capturing the function of each gossip event were not mutually exclusive. Instead, each gossip event was annotated in a multiple-choice manner. Based on existing literature (e.g., Beersma and van Kleef, 2012; Grosser et al., 2012; Dores Cruz et al., 2019), we distinguished between six different gossip functions: (1) information sharing, (2) enforcing group norms and values, (3) group protection, (4) entertainment, (5) emotion venting, and (6) negative influence. The code *information* includes both the exchange and validation of information (Foster, 2004; Beersma et al., 2019). The code *group norms* captures gossip behavior that establishes and maintains work-related group norms and values (Baumeister et al., 2004; Foster, 2004). The code *group protection* captures gossip that protects the recipient(s) from free-riders (Dunbar, 2004; Beersma and van Kleef, 2012). Due to the setting of our study, we also used this code when participants gossiped about unpleasant people such as angry patients or hostile visitors. Gossip for entertainment purposes is captured by the code *entertainment* and consists of a funny or humorous exchange (Foster, 2004; Grosser et al., 2012) in which the gossiper and recipient(s) are pleasantly amused. Gossip statements to vent emotions are characterized by negative emotions of the gossiper such as stress, anger, concerns, or disappointment (Waddington, 2005; Altuntaş et al., 2014; Dores Cruz et al., 2019). This is captured with the code *emotion venting*. The category *negative influence* is characterized by malicious opinions about non-allies (Beersma et al., 2019) and the gossiper’s intent to convince the recipient(s) to revise their opinion of the target, sometimes to enhance their own status (Grosser et al., 2012).

### Annotation Approach

We annotated gossip behavior and focused on verbal behavior only. Three independent raters used INTERACT software (Mangold, 2021) and our refined gossip coding scheme manual to annotate the recordings, following a two-step procedure. All annotations were based on the real-time recordings in order to be able to pay attention to vocalizations and tone of voice (e.g., to catch gossip that was meant in a sarcastic way). The data was therefore not transcribed, except for the anonymized examples given for illustrative purposes below. Note that we translated those examples into English, as the original meetings were conducted in German. Moreover, because workplace gossip is a context-embedded phenomenon (Mills, 2010), information about the (conversational) context is crucial to correctly understand and categorize gossip behavior. Thus, all recordings were coded in correct order instead of parsing the data into randomized single events.

As a first step, we established agreement on correctly recognizing gossip as such. One of the raters unitized the entire flow of communication into sense units (Bales, 1950). Sense units are the smallest speech segments that express a complete thought, which enables a very fine-grained analysis. A sense unit usually corresponds to a single statement. For each unitized event, the onset and offset time and speaker were saved. As the sense units were segmented based on content, the duration of the sense units varied considerably from 0.13 to 51.87 seconds (*M* = 4.35, *SD* = 4.37). Then, the first and second rater independently classified each sense unit according to whether it contained a gossip statement or not. As soon as the speaker talked about an absent person, this was annotated as gossip; everything else was annotated as *other*. The unitizing of gossip into individual gossip events is in line with common conceptualizations of workplace gossip, as gossip does not necessarily require back- and forth communication, but instead can be made of a single, unidirectional statement (Brady et al., 2017). All eight meetings were analyzed by both raters. Like Robbins and Karan (2020) and following suggestions by Klonek et al. (2020), we used intraclass correlation coefficients (ICC) as a measure for inter-rater agreement. We calculated a two-way random absolute average measures ICC (McGraw and Wong, 1996) across all meetings to check agreement on whether gossip events were recognized as such (ICC = 0.99). As interrater reliability was excellent (Cicchetti, 1994), we proceeded with the next step.

The second step involved a detailed annotation of each gossip event. Doing so, the first and third rater annotated each gossip event according to its valence and functions. All eight recordings were analyzed by both raters. Again, we calculated two-way random absolute average measures ICCs across all meetings. ICC scores ranged from 0.88 to 1.0, which indicates excellent inter-rater agreement (Cicchetti, 1994).

## Results

In total, our sample comprised 4,804 sense units at the behavioral event level, of which a total of 626 events comprised gossip. On average, gossip statements accounted for 12.9% of all behavioral events observed per meeting. Notably, all observed gossip statements were work-related and concerned absent third persons in the organization.

### Distribution and Expressivity of Gossip Valence

Regarding our first research question (i.e., how the valence of gossip unfolds in the flow of conversation), we first calculated the averaged distribution of the observed gossip. On average, nearly half of the observed gossip events were neutral (45.7%), followed by negative (27.2%), ambiguous (20.1%), and positive gossip (7%). As we were also interested in differences depending on the meeting length, we compared the distribution of gossip statements across the eight teams in Table 2. In three of the eight team meetings (Teams 1, 3, and 7 in Table 2), more neutral gossip was observed compared to the other three gossip types (ranging from 58.1% to 70%). Interestingly, those three meetings were generally shorter (ranging from 17 to 29 min), indicating that meeting length might be related to gossip valence. Turning to the evaluative gossip statements, our analysis revealed that negative gossip events occurred a lot more frequently than positive gossip. An exception is Team 1, as not a single negative gossip event was recorded in this meeting. However, the proportion of ambiguous gossip in this team was relatively high (25%), i.e., gossip that could not be clearly evaluated as positive or negative but was of an evaluative nature. Overall, all teams showed a moderately high proportion of ambiguous gossip (ranging from 16.3% to 27.4%), suggesting that gossip often occurs “between the lines” and cannot be clearly categorized as positive or negative (at least by an outside observer). In all of the observed meetings, the gossip switched from one valence to another, rather than being exclusively of a similar valence. For example, when talking about the same target, the same speaker could alternate between positive and negative gossip within a single gossip episode (e.g., “*We must be glad that someone is coming*… [positive] *She may perhaps be as she is*… [negative] *but we have the chance to take her in again.* [positive]”).

**TABLE 2:** Distribution of gossip events per team meeting.

Team	*N*	Meeting length	Gossip (%)			
			Neutral	Negative	Positive	Ambiguous
Team 1	7	29	70.0	/	5.0	25.0
Team 2	9	39	33.3	46.7	8.0	12.0
Team 3	6	17	58.1	14.0	7.0	20.9
Team 4	9	64	38.7	29.0	4.8	27.4
Team 5	10	49	36.8	34.9	4.7	23.6
Team 6	7	58	35.2	34.7	8.5	21.6
Team 7	8	17	65.5	17.2	3.5	13.8
Team 8	6	73	28.3	41.3	14.1	16.3

*Meeting length in minutes. Relative frequencies of gossip events, reported in percent. *N* = number of nurses per meeting.*

**FIGURE 2:**
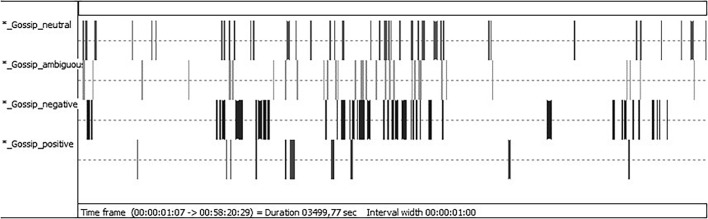
Time line chart illustrating the flow of gossip events over the course of one sample meeting (generated with INTERACT software). The entire meeting is shown (approx. 58 min in total). The black and gray marks indicate that a gossip event was recorded. The first row shows instances of neutral gossip, the second row shows instances of ambiguous gossip, the third row shows instances of negative gossip, and the fourth row shows instances of positive gossip. The wider the marks, the longer the event was recorded (duration).

To further illustrate how dynamically team members switched between the different gossip types over the whole course of a meeting, Figure 2 shows the stream of coded gossip statements in one sample meeting (Team 6). This particular meeting lasted for 58 min and comprised a total of 199 gossip events. Most gossip was observed in the middle of the meeting. Here, gossip events of different valence seemingly occurred almost simultaneously. That is, team members rapidly alternated between neutral, negative, ambiguous, and, rarely, positive gossip events. Neutral, ambiguous, and positive gossip appeared to be more evenly dispersed throughout the meeting, whereas negative gossip was more clustered and occurred in a few, concentrated episodes.

In order to gain insights into the alternation of different types of gossip behavior, we further analyzed sequences of gossip events on a smaller scale. We conducted lag sequential analyses at lag1 (i.e., from one statement to the immediate next) and lag2 (i.e., from a given behavior to the next-but-one behavior in the interaction stream). We focused on the conditional probabilities of one specific gossip behavior following another. To test whether the observed behavioral patterns of gossip at lag1 and lag2 were statistically meaningful, we calculated z-values. The results are presented in Table 3. Each type of gossip behavior (neutral, negative, positive, and ambiguous) was significantly followed by the same gossip behavior (e.g., negative gossip is significantly followed by negative gossip), indicating a mostly self-sustaining pattern.

**TABLE 3:** *Z*-values of conditional probabilities of gossip behavior sequences at lag1 and lag2.

Preceding gossip behavior (*Lag0*)	Following gossip behavior							
	Neutral		Negative		Positive		Ambiguous	
	*Lag1*	*Lag2*	*Lag1*	*Lag2*	*Lag1*	*Lag2*	*Lag1*	*Lag2*
Neutral	6.80*	3.28*	−5.99*	−3.80*	–1.09	0.11	–0.46	0.42
Negative	−5.47*	−3.56*	7.79*	5.38*	–1.62	–0.97	–1.47	–1.38
Positive	–1.66	0.47	–1.33	–1.01	4.64*	2.91*	0.48	–1.34
Ambiguous	–0.71	–0.12	–1.02	–1.03	0.13	–0.96	1.97*	2.01*

Significant sequential effects for lag1 (immediate next behavior) and lag2 (next-but-one behavior) are indicated by *z*-values larger than 1.96 or smaller than −1.96 marked with *.

### Functions and Valence of Workplace Gossip

Regarding our second research question, we now turn to the linkages between gossip valence and the social functions associated with each gossip event. An overview is provided in Table 4. To recall, each gossip event could only be assigned to one of the four valence categories. However, several social functions could be assigned simultaneously to the same gossip event. On average information exchange was the most frequent function of the observed gossip statements (98.8%), followed by group norms and values (34.2%), emotion venting (29.1%), negative influence (12.1%), and group protection (9.9%). The least frequent function was entertainment (4.7%).

**TABLE 4:** Average percentages of gossip functions for each valence.

		Function					
		Information	Group norms and values	Group protection	Entertainment	Emotion venting	Negative influence
Valence	Positive	**100%** *“Everybody likes him.”*	**35.0%** *“So she prepares food and stuff and I think that helps a lot.”*	/	**0.7%** *“We really all got along well. He was - he is a very sweet person.”*	/	/
	Negative	**98.0%** *“Well, certainly some relatives come infrequently, no question.”*	**43.8%** *“For example, A. is not so good at writing reports.”*	**31.2%** *“With the exception of Mr. B., this can also be handled quite well.”*	**9.1%** *“She almost sleeps sitting up.”*	**74.7%** *“She’s just ranting.”*	**36.4%** *“I wanted to clear this up with C., but. no chance.”*
	Ambiguous	**99.5%** *“Um, I think, F. is generally open for that. She is scared.”*	**40.9%** *“But then she also has to get used to it. She has to work her hours, because she is employed full-time.”*	**5.6%** “*Because going to the toilet with her during the day is not really possible, right?”*	**12.5%** *“But a married couple will be quite nice again, if the husband also keeps up, right?”*	**43.6%** *“But she really does go to the bathroom 50 times a day on a regular basis.”*	**7.1%** *“With U. we never know if she will come back. She now simply has to sign up for the days off. It’s the way it is.”*
	Neutral	**99.0%** *“The coughing has nothing to do with the food, it’s chronic.”*	**25.9%** *“Mrs. M. can also be washed in good time, i.e., at 6:30 a.m. She’s already awake by then.”*	/	/	/	/

*The table shows the averaged occurrence and distribution of each function (column) depending on the valence of the gossip event (row). Each gossip event could only be assigned to one of the four valence categories. The functions were not mutually exclusive so that multiple functions could be assigned to the same gossip event. Therefore, the presented percentages (in bold) do not necessarily add up to 100% per row.*

Neutral gossip events served the fewest different social functions. Specifically, of the six different social functions described in the literature, neutral gossip events only fulfilled the two functions of information sharing and establishing and maintaining work-related group norms and values. A comparison of the two functions shows that information sharing was noticeably more often associated with neutral gossip than the group norm function. Positive gossip events were the least frequent ones and, apart from information sharing, were only used to establish and maintain work-related group norms and values, and in very few cases, to provide (positive) entertainment. Negative and ambiguous gossip showed the most diverse pattern. All six social functions were assigned to these two gossip types. Starting with negative workplace gossip events, we found that information sharing and emotion venting were the two social functions most frequently associated with this type of behavior. The least frequently used function of negative gossip was entertainment. A slightly different pattern of results was found for ambiguous gossip. Ambiguous gossip was most frequently associated with information sharing, followed by emotion venting, enforcing group norms and values, entertainment, and group protection and negative influence.

Particularly noticeable when looking at the functions is the very frequent occurrence of the information sharing category. Nearly all gossip events served to share information, regardless of their valence, and were often accompanied by additional functions. Gossip events that only served as information sharing were neutral and seemed to mostly concern relevant information about patients (e.g., *“Because she is completely confused. She is simply at high risk of falling”; “Ms. F will move to room X.”*) and only rarely concerned information about colleagues (e.g., “*The kitchen would like to have some feedback every two weeks.*”).

### Ancillary Observations

Beyond the findings related to our two research questions, we share further observations regarding (1) the general targets of workplace gossip, (2) the relationship between the valence of gossip statements and its linguistic expressions, and (3) the relationships between the content of gossip statements and their social functions.

#### General Targets of Gossip

Generally, workplace gossip appeared to be not just about a specific target (i.e., scapegoats) or a type of person (e.g., patients), but rather about any work-related persona. Targets of gossip included (1) supervisors (*“This is not Mr. S.’s or Ms. M.’s personal idea, but they have to.”*), (2) team members (“*She arrived half an hour late today and leaves on time”)*, (3) colleagues from other departments (“*Because, everyone will read this. Then, S. will come, then R. will come, they read this and then, in the smokers’ corner go ‘mh mh mh’ [mimics gossiping].”)*, (4) external colleagues such as physiotherapists or physicians (“*Yes, well, that’s my personal feeling, I think he could have waited a little longer, because he’s done enough dirty work here. So, he’s billing for prescriptions that didn’t go that way, period.*”), (5) patients (“*Because B., you know yourself, he is not always so good to handle, so you might need a second one [nurse].”*), and (6) relatives of patients (“*Only, um, the daughter finds fault with everything.*”).

#### Relationship Between Gossip Valence and Linguistic Expression

Among further qualitative observations, we noticed that when gossip was clearly negative, it was usually accompanied by a particular linguistic expression. In our sample, we observed that gossip senders seem to often use lexical hedges when they were clearly speaking negatively about someone. Rather than expressing certainty or decisiveness, lexical hedges express tentativeness, convey vagueness, and can be used to soften a statement (Lakoff, 1973). Examples from our data include “*that’s my personal feeling.”, “I think.”, “I don’t want to badmouth*…*, but.”*, or “*I don’t want to talk behind her back, but*…*”.* A sample negative gossip event including this linguistic style was: *“So I don’t want to badmouth the care assistants, no, I’m not a care assistant myself and I—that’s what we all criticize actually—the care assistants who are here in the house, not in the whole house, I don’t know, I don’t want to attack anyone personally, but they don’t do a decent job, I’m sorry.”*

#### Relationship Between Gossip Content and Functions

Gossip events used to vent emotions were apparently not restricted to a specific target group but concerned a broad range of target groups: (1) hard-to-handle patients (e.g., aggressive personality, e.g., “*She’s just ranting.*”), (2) disrespectful relatives of patients (e.g., being overly controlling, e.g., “*Only, um, the daughter finds fault with everything.*”), (3) low-performing colleagues (e.g., doing a sloppy job, using up all utensils but not ordering new ones, e.g., “*And then we’re standing there having dinner. No cup, no plate*.”), (4) colleagues with poor work ethic (e.g., coming in late, only doing the bare minimum, e.g., “*Instead of half past six, he came at seven.*”), and (5) the general management (e.g., not hiring adequate personnel, e.g., “*Mr. S., for example - I’m not criticizing Mr. S. or the house, but in general now. They have also started to criticize that we have, for example, too many people who can only do early shifts. [*…*] And yet, they always hire early shifts.*”).

Statements to establish and maintain work-related group norms and values seemed to have often been about how to best treat patients or general morale. Often, those statements appeared to have a “hidden” gossip target, besides the obvious subject of the statement. For example, the exact wording of one gossip statement was “*Mrs. H., Mrs. B., it can’t be that they have dirty sheets in bed for days on end.*” In terms of content, the two patients Mrs. H. and Mrs. B. were mentioned here. However, the hidden target seemed to be a team member who did not change the bed sheets.

Gossip to protect the group appeared to concern (1) free-riding colleagues (e.g., external colleagues who are billing work they never did, e.g., *“D. is also one of those issues. We have to keep a very close eye on him now.”*), but also (2) patients (e.g., patients that are aggressive or hard to handle, e.g., “*I think Mrs. I. is difficult.*”), or (3) their relatives (e.g., relatives that are overly critical, racist, or controlling, e.g., *“But to come back to C., she is currently very unhappy.”).* In those cases, the team members appeared to use gossip to warn each other about the targets—to be prepared for possible negative encounters or to avoid certain people.

Ambiguous gossip statements that were used to entertain seemed to often include sarcasm (e.g., answering with “*just before the start of vacations*” after being asked when a colleague who is mostly absent and disengaged will come back to work again).

Gossip that was used to negatively influence the others’ opinion of the target appeared to be mostly about colleagues that were doing a poor job. Other statements often concerned colleagues that were having an extremely poor work ethic and would classify as the typical free-rider (e.g., *“So it’s definitely been the case that he kind of had signatures [for appointments] where he wasn’t there.”).*

## Discussion

This study set out to empirically investigate gossip behavior in the field. Using quantitative interaction analysis, we explored workplace gossip as a dynamic communication event in conversations. In particular, with two research questions, we analyzed (1) how the valence of workplace gossip unfolds in the flow of communication, and (2) how the social functions and valence of gossip statements interact in conversations. To do so, regular team meetings of eight elderly care teams were investigated. The results show that workplace gossip is a highly contextualized and dynamic conversational event that is embedded in the flow of communication. Concerning our first research question, we found that conversational workplace gossip events are dynamic and quickly change back and forth in valence over the course of the conversation. When considering the entire flow of conversation in a team meeting, it appears that negative workplace gossip is not evenly distributed throughout the conversation. Rather, unlike other types of gossip events (i.e., neutral, ambiguous, and positive), negative gossip appears to happen in more clustered, focused, and longer conversational episodes. Lag sequential analyses further revealed self-sustaining behavioral patterns for each type of gossip, such that a particular type of gossip tends to trigger more of the same (e.g., negative gossip is followed by more negative gossip). Regarding our second research question, our findings indicate that workplace gossip serves a variety of social functions simultaneously. As the valence of the gossip events changes, the functions of gossip change accordingly. These findings underscore the importance of considering and analyzing workplace gossip as a dynamic conversational event.

### Theoretical Implications

The amount of workplace gossip in the meetings observed in this study corresponds to the proportion of general gossip in every-day conversations (Robbins and Karan, 2020). Almost half of the gossip we observed was neutral, which is likely caused by the special setting and sample of meetings in elderly care. As nurses face high workloads, they do not always get the chance to exchange all information that need to be shared even when they are working in the same shift (Testad et al., 2010; Zhang et al., 2014). Therefore, besides various other reasons, meetings are held for exchanging work-relevant information (Allen et al., 2015). Naturally, in health care this includes (neutral) information about other absent people such as patients (Kerr, 2002), which falls under the definition of (neutral) gossip (Brady et al., 2017). A multi-sample study investigating every-day gossip by Robbins and Karan (2020) shows similar results regarding the neutral valence of gossip. Their findings point out that most of the informal everyday gossip is neutral instead of negative or positive. Interestingly, they found an even greater proportion of neutral gossip compared to our results, even though we investigated a special sample whose job requires them to share (neutral) information about others.

The special role of neutral gossip is further highlighted by its linkage to the social functions of gossip. Our findings indicated that neutral workplace gossip mostly fulfilled the function of information sharing and was only rarely used to establish and maintain work-related norms and values. This raises the conceptual question whether neutral workplace gossip in information-oriented meetings can be regarded as gossip as we understand it, i.e., as informal and evaluative talk about absent targets (Brady et al., 2017). Neutral gossip in the observed context of information-oriented meetings was not spontaneous but appeared to be part of the formal meeting agenda (e.g., discussing the treatment of a patient). Thus, it could be argued that the neutral gossip in our sample did not have any of the defining features of workplace gossip (i.e., being informal and evaluative) except that it was talk about absent targets. Moreover, neutral gossip did not seem to serve any social functions characteristic of workplace gossip other than (normative) information sharing and enforcement of group norms, suggesting that this is a less consequential behavior. Further naturalistic studies should explore this question in more depth. In combination with our results, this could help to sharpen the focus on what gossip actually entails and contribute much needed construct clarity.

Furthermore, we observed a high amount of what we now call “gossip expressivity.” That is, team members alternated between different types of gossip much more frequently than would be expected by previous theory. In other words, even when talking about the same target, the same gossiper could alternate between two opposing evaluations (i.e., positive and negative). This raises the question whether it makes sense at all to look at different gossip types in isolation, for example when considering how gossip relates to meeting and team outcomes. Previous studies compared negative vs. positive workplace gossip as a whole and found controversial effects regarding the valence of gossip (cf. Grosser et al., 2012; Kuo et al., 2018). Providing a more nuanced picture, our findings indicate that a “gossip conversation” is not only negative or only positive but includes much more complex and dynamic gossip expressions. In fact, we conclude from our findings that workplace gossip should be considered in terms of specific conversational events, instead of an entire gossip conversation. Failure to distinguish between different types of dynamic gossip expressions throughout a conversation may explain previous mixed results regarding the influence of the valence of gossip. By aggregating the whole conversation as being of only one particular valence, potentially different effects of different nuances of gossip expressions remain uncovered.

Regarding the structural embeddedness of different gossip types, our results suggest self-sustaining patterns of each gossip type. When looking at the entire stream of conversation, specifically negative gossip seems to happen in more condensed clusters, illustrating the self-sustaining micro-sequences of (negative) gossip on larger scale. Negative gossip was often used for emotion venting and includes negative evaluations of others, indicating conceptual overlaps with complaining behavior (cf. Kauffeld and Meyers, 2009). Interestingly, in a study by Lehmann-Willenbrock et al. (2011), complaining behavior showed similar self-sustaining patterns, in terms of complaining circles observed during team meetings. The clustered pattern of both gossip and complaining might be due to a common denominator of these two behaviors, namely negative valence and the potential perception of a deviant behavior. Depending on the context, both negative gossip and complaining can either be perceived as a socially undesirable behavior, or it may open a (temporally limited) safe space for others to join in the behavior. Future studies can clarify this by observing and comparing emergent behavioral patterns surrounding other types of negatively valenced behavior (e.g., negative humor).

Our findings also have implications for linguistic research on gossip. We noticed that negative gossip events were often characterized by the use of linguistic hedges (Lakoff, 1973), meaning that, apparently, negative gossip was often embedded in phrases to leverage or justify the negative evaluation. This indicates that, at least in more formal setting such as regular team meetings, negative gossip is a much more delicate communicative behavior than previously assumed. Instead of using direct language to criticize absent third parties, the participants in our sample were seemingly concerned with politeness. This subtlety of gossip also leads to implicate that when not paying close attention to the occurrence of gossip in the moment, some gossip statements may not be recognized as such. As a consequence, the recognition and recollection by participants could be biased and self-reported results distorted.

Additionally, our study demonstrates that the functions of gossip and the valence of gossip appear to be linked. The distribution of the functions depending on the valence of workplace gossip in our data indicates that half of the characteristic functions of gossip (i.e., group protection, negative influence, and emotional venting) are only applicable to negative or ambiguous gossip, but not to positive or neutral gossip. Thus, only negative and ambiguous gossip statements served any of the six functions. Interestingly, apart from information exchange, the majority of negative and ambiguous gossip observed in our study was used as an outlet for negative emotions. This finding is consistent with previous research showing that emotion venting is one of the most important motives for nurses to gossip (e.g., Waddington and Fletcher, 2005; Altuntaş et al., 2014). Workplace gossip, in this case, may just be a symptom of the stressful work environment and the result of having no other alternative to cope with the negative emotions associated with work stress. This underlines the importance of the context when investigating gossip.

Finally, our findings provide insights into the complexity of the content of workplace gossip statements. As the gossip statements can fulfill multiple functions simultaneously, the target of the gossip statement can also be more than one person, and even the valence can be a mix of positive and negative evaluations (i.e., ambiguous gossip). Thus, saying one thing can mean multiple things to multiple people. Non-verbal and paralinguistic cues such as facial expressions (saying something “positive” but rolling the eyes) or tone of voice (the literal words are kind, but the tone of voice is aggressive) add multiple layers to the gossip statement which alternate its meaning. Consequently, accounting for situational variables, even on a micro level, is necessary to unravel the characteristics and effects of workplace gossip.

### Limitations and Future Research

As with any exploratory work, our findings should be interpreted with caution. Whereas the sample size was relatively large at the behavioral event level (*N* = 4,804), which served to address our focal research questions, the number of observed teams and meetings was small (*N* = 8). Further, we observed gossip behavior in a very specific setting and sample, which precludes generalization of our findings to varying contexts of workplace gossip. In order to validate our gossip coding scheme and obtain more reliable and generalizable results, future studies should capture more heterogeneous samples and different contexts.

One context that is distinct from the current study but would be of particular interest to address in future work concerns online team interaction settings such as virtual or hybrid meetings (Blanchard, 2021). These (partly) virtual settings may hinder workplace gossip occurrences as communication side channels are rare or transferred to other means such as chat. The recent work by Blanchard (2021) shows that currently, informal communication is generally neglected in virtual meetings. Consequently, virtual meetings are also missing out parts of the social functions of informal communication. Future studies on workplace gossip in remote settings may also provide implications on how to best design future hybrid and virtual meetings in order to still support informal communication among colleagues.

By observing meetings in the most naturalistic setting possible and reducing the salience of the recording (i.e., using only a single camera with built-in microphone), we aimed to reduce the risk of social desirability. Despite being regarded as deviant behavior (Foster, 2004), we observed a broad range of negative workplace gossip similar to a previous observation study on workplace gossip in meetings (Hallett et al., 2009). While we interpret this finding as a hint that social desirability was less of a concern, we cannot definitely rule out social desirability bias in the observed meeting behavior. Future observational studies on workplace gossip might add a social desirability survey (e.g., Clancy and Gove, 1974) and explore to what extent individual attendee behavior correlates with such a measure.

Finally, a considerable setback of our methodology concerns the substantial annotation effort, which is time consuming, labor intensive, and to most scholars—understandably—rather unattractive (Kolbe and Boos, 2019). Moreover, despite exhaustive training, human annotators might still be susceptible to biases and, for example, tend to associate a person they do not perceive as likeable with negative gossip. To validate the human annotations, future studies could compare them with additional behavior-based measures. For example, when analyzing transcribed conversations, the annotated valence of workplace gossip statements could be compared against the results of text analysis using established tools such as LIWC (Pennebaker et al., 2015).

In general, we encourage future studies on workplace gossip to employ more behavioral observation measures in order to capture the complexity, temporal dynamics, and nuances of this phenomenon. By investigating gossip behavior *in situ*, behavioral observation approaches allow for an array of analyses that can account for the temporal and structural embeddedness of gossip in social interactions as well as consider various behavioral modalities (e.g., verbal, para-linguistic, non-verbal).

### Implications for Interdisciplinary Research Collaborations

Interdisciplinary collaborations of social scientists and computer scientists would allow to follow multimodal approaches when analyzing workplace gossip. Considering the technological advancements within the past years such as automated facial expression (e.g., Kudiri et al., 2016) or automated speech recognition (e.g., Milde et al., 2021), future research could identify non-verbal and paralinguistic cues of workplace gossip valence. In our context, we focused primarily on verbal behavior. However, by annotating the audio-recorded spoken words instead of transcripts, paralinguistic cues such as tone of voice were implicitly taken into account as well. A mismatch between the literal spoken words and tone of voice even were a characteristic for ambiguous workplace gossip, increasing the level of difficulty to correctly detect the valence of the gossip statement. Future interdisciplinary work between social science and computer science could advance workplace gossip research by explicitly focusing on paralinguistic cues in combination with lexical features to detect the underlying valence of gossip statements (see Aldeneh et al., 2017, on the prediction of valence using lexical and acoustic features).

As reliable and innovative annotation systems are important for both social and computer scientists (Allen et al., 2017), interdisciplinary collaborations between the two research areas also have the chance to offer ground-breaking implications and solutions that advance research in both areas (Beck et al., 2017; Lehmann-Willenbrock et al., 2017). Future interdisciplinary collaborations could, for example, improve the extensive human-powered annotation processes by supporting the manual work with automated AI/ML annotation processes. Our fine-grained analysis and dataset can provide a starting point for gathering larger corpora and potentially developing automated annotation systems for capturing gossip in the future.

## Data Availability Statement

The datasets presented in this article are not readily available because of data confidentiality. The anonymized annotations of the observed interaction data (but not the raw behavioral data in any form), can be provided by the first author upon request. Requests to access the datasets should be directed to VB, vanessa.begemann@uni-hamburg.de.

## Ethics Statement

The studies involving human participants were reviewed and approved by Local Ethics Committee, Faculty of Psychology and Human Movement Science, University of Hamburg. The patients/participants provided their written informed consent to participate in this study.

## Author Contributions

VB developed the idea for the manuscript, took the led in writing, performed the analyses, and interpreted the data. SL developed the idea for the manuscript, collected the data, and contributed to writing the manuscript. AM and FS critically revised the manuscript for intellectual content and contributed to writing the manuscript. NL-W obtained funding, led the research project, critically revised the manuscript for intellectual content, and contributed to writing the manuscript. All authors approved the manuscript to be published.

## Conflict of Interest

The authors declare that the research was conducted in the absence of any commercial or financial relationships that could be construed as a potential conflict of interest.

## Publisher’s Note

All claims expressed in this article are solely those of the authors and do not necessarily represent those of their affiliated organizations, or those of the publisher, the editors and the reviewers. Any product that may be evaluated in this article, or claim that may be made by its manufacturer, is not guaranteed or endorsed by the publisher.
